# Occurrence of hypertension during third‐line anlotinib is associated with progression‐free survival in patients with squamous cell lung cancer (SCC): A post hoc analysis of the ALTER0303 trial

**DOI:** 10.1111/1759-7714.14076

**Published:** 2021-07-17

**Authors:** Jianhua Shi, Guimin Chen, Haitao Wang, Xiuxiu Wang, Baohui Han, Kai Li, Qiming Wang, Li Zhang, Zhehai Wang, Ying Cheng, Jianxing He, Yuankai Shi, Weiqiang Chen, Yi Luo, Lin Wu, Xiuwen Wang, Kejun Nan, Faguang Jin, Jian Dong, Baolan Li, Zhian Liu

**Affiliations:** ^1^ Department of Oncology Linyi Cancer Hospital Linyi China; ^2^ Department of Cardiothoracic Surgery Zhejiang Provincial People's Hospital, People's Hospital of Hangzhou Medical College Hangzhou China; ^3^ Department of Pulmonary Medicine Shanghai Chest Hospital, Shanghai Jiaotong University Shanghai China; ^4^ Department of Thoracic Oncology Tianjin Medical University Cancer Hospital Tianjin China; ^5^ Department of Internal Medicine Affiliated Cancer Hospital of Zhengzhou University Zhengzhou China; ^6^ Department of Internal Medicine Henan Cancer Hospital Zhengzhou China; ^7^ Department of Respiratory Diseases Peking Union Medical College Hospital Beijing China; ^8^ Department of Internal Medicine‐Oncology Shandong Cancer Hospital Jinan China; ^9^ Department of Thoracic Oncology Jilin Cancer Hospital Changchun China; ^10^ Department of Thoracic Surgery The First Affiliated Hospital of Guangzhou Medical University Guangzhou China; ^11^ Department of Medical Oncology Cancer Hospital Chinese Academy of Medical Sciences Beijing China; ^12^ Department of Pulmonary Medicine Lanzhou Military General Hospital Lanzhou China; ^13^ Department of Medical Oncology Hunan Cancer Hospital Changsha China; ^14^ Department of Chemotherapy Qilu Hospital of Shandong University Jinan China; ^15^ Department of Oncology The First Affiliated Hospital of Xi'an Jiaotong University Xi'an China; ^16^ Department of Respiratory Diseases Tang Du Hospital Xi'an China; ^17^ First Department of Medical Oncology Yunnan Cancer Hospital Kunming China; ^18^ Department of General Medicine Capital Medical University, Beijing Chest Hospital Beijing China

**Keywords:** anlotinib, hypertension, lung cancer, squamous cell carcinoma, survival

## Abstract

**Background:**

There is a lack of targeted therapeutic options for squamous cell lung cancer (SCC). Accelerated hypertension is an issue with many targeted therapies for lung cancer. This study aimed to analyze the efficacy of anlotinib, based on progression‐free survival (PFS) and overall survival (OS) in patients with SCC, stratified by hypertension and Eastern Cooperative Oncology Group (ECOG) score.

**Methods:**

This was a post hoc analysis of a multicenter, double‐blind, phase III ALTER0303 randomized controlled trial. Only patients with SCC were included. The occurrence of hypertension during the study period was defined according to CTCAE 4.03. OS and PFS were the primary and secondary endpoints, respectively. The patients were stratified according to hypertension and ECOG score, respectively.

**Results:**

The median PFS in the patients who developed hypertension was longer than in those who did not (7.2 (95% CI: 3.5–11.0) versus 3.2 (95% CI: 1.2–5.3) months, *p* = 0.001; HR (95% CI), 0.4 (0.2–0.8)). In the ECOG 0 patients, the median PFS in the patients who developed hypertension versus those who did not was 5.6 vs. 1.8 months, respectively (Figure 2(d)). In the ECOG 1 patients, the median PFS in the patients who developed hypertension versus those who did not was 7.0 (95% CI: 3.0–11.0) vs. 4.8 (95% CI: 1.2–8.5) months (*p* = 0.043). No statistically significant differences were found in OS in the stratified analyses.

**Conclusions:**

The occurrence of hypertension might be a clinical indicator predicting the efficacy of third‐line anlotinib treatment in patients with SCC.

## Graphical abstract

In SCC patients who received third‐line anlotinib treatment, the median PFS was longer in patients who developed hypertension than in those who did not. Our results suggest that the occurrence of hypertension during treatment might be a clinical indicator predicting the efficacy of third‐line anlotinib in patients with SCC.

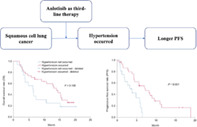

## INTRODUCTION

Lung cancer is the leading cause of cancer‐related death worldwide.^1^, ^2^ Non‐small cell lung cancer (NSCLC) accounts for approximately 85% of newly diagnosed lung cancer cases, and about 20%–30% of all NSCLCs are squamous cell lung cancer (SCC).^3^ With the scientific advances on specific molecular alterations in tumors, small‐molecular targeted therapies have been developed against NSCLC.^4^, ^5^, ^6^ Unfortunately, the available agents are still poorly effective for SCC treatment because this subset of lung cancer is typically diagnosed at an advanced stage and often does not display targetable genetic alterations.^7^ At present, platinum‐based chemotherapy is commonly used against SCC, and the median overall survival (OS) and progression‐free survival (PFS) of patients with advanced SCC are only 10 and 5.6 months, respectively.^8^

Because angiogenesis plays a crucial role in tumor growth,^9^ angiogenesis inhibitors have been evaluated in treating patients with NSCLC,^10^ but few data are available for patients with SCC. Anlotinib is a novel small molecule tyrosine kinase inhibitor targeting the vascular endothelial growth factor receptor (VEGFR), fibroblast growth factor receptor (FGFR), platelet‐derived growth factor receptor (PDGFR), and c‐Kit.^11^, ^12^ It has been approved as a third‐line treatment for refractory advanced NSCLC by the China Food and Drug Administration (CFDA) on May 9, 2018.^13^ A previous phase II trial (ALTER0302) showed a better PFS in patients with advanced NSCLC treated with anlotinib compared with placebo (4.8 vs. 1.2 months, *p* < 0.0001).^14^ In the ALTER0303 phase III trial, both the OS and PFS of patients with advanced NSCLC were significantly longer in the anlotinib group (median, 9.6, and 5.4 months) than those in the placebo group (median, 6.3 and 1.4 months).^14^ Moreover, anlotinib also displays manageable toxicity, long circulation, and broad‐spectrum antitumor potential.^15^, ^16^ Most SCC patients are diagnosed at a poor condition and display fewer genetic target, and thus the benefit of treatments are limited.^17^, ^18^, ^19^ Because of the lack of recommended third‐line drugs with therapeutic effect for patients with SCC, it is worth examining the efficacy and safety of anlotinib in this subtype of NSCLC.

Angiogenesis inhibitors can impact normal signaling in healthy tissues, leading to serious toxicities and adverse outcomes such as accelerated hypertension.^20^ Hypertension is a common adverse event related to antiangiogenic therapy. Several studies have demonstrated that the development of hypertension during the VEGF‐target therapy is associated with the clinical outcome of NSCLC^21^, ^22^ and several other cancers.^23^, ^24^, ^25^ Hypertension has been suggested as a marker of efficacy in patients with renal cell carcinoma treated with sunitinib.^26^ However, whether the incidence of hypertension will influence the benefits of anlotinib on SCC patients remains unknown.

Based on the ALTER0303 phase III trial results, this study aimed to analyze the efficacy of anlotinib using Cox proportional hazards regression analysis and Kaplan–Meier curves for predicting the PFS and OS of patients with SCC, stratified by hypertension and Eastern Cooperative Oncology Group (ECOG) score.

## METHODS

### Study design and patients

This study was a post hoc analysis of the multicenter, double‐blind, phase III ALTER0303 randomized controlled trial.^14^, ^16^ In the original trial, patients with NSCLC were enrolled from 31 hospitals in China between March 1, 2015, and August 31, 2016. In the original trial,^14^, ^16^ the eligibility criteria for patients were: (i) should be between 18–75 years of age, (ii) histologically confirmed with NSCLC, (iii) life expectancy of ≥3 months, and (iv) progression after at least one line of chemotherapy and one line of targeted therapy for patients with driver mutations, or after at least two lines of chemotherapy for patients without driver mutations. The exclusion criteria were: (i) hemoptysis (>50 ml/day), (ii) centrally located SCC with cavitary features, (iii) symptomatic brain metastases or brain metastases controlled for less than two months, or (iv) systemic antitumor therapy scheduled in the preceding four weeks or during the trial. All patients received oral anlotinib (12 mg/day on days 1–14 of a 21‐day cycle.) (Chia Tai Tianqing Pharmaceutical Group Co., Ltd) or placebo (Chia Tai Tianqing Pharmaceutical Group Co., Ltd) until progression, unacceptable toxicity, withdrawal of patient consent, or death. The study was approved by all participating centers. All patients provided their informed consent. The study was registered (ClinicalTrials.gov identifier: NCT02388919).

For this post hoc analysis, only patients with SCC were included. In addition, patients with poor blood pressure at baseline were also excluded (i.e., systolic blood pressure ≥ 150 mmHg and/or diastolic blood pressure ≥ 100 mmHg). The occurrence of hypertension during the study period was defined according to CTCAE 4.03. According to CTCAE, hypertension is a continuous increase in blood pressure that does not fluctuate with drug administration and occurs during the entire medication period. Hypertension was defined as a clinical syndrome characterized by increased systemic arterial blood pressure (systolic blood pressure ≥ 140 mmHg and/or diastolic blood pressure ≥ 90 mmHg), which may or may not be accompanied by functional or organic damage to the heart, brain, kidney, and other organs.

### Outcomes and definitions

OS and PFS were the efficacy assessments in this post hoc study. The OS was defined as the time from randomization to death or the last follow‐up. The PFS was defined as the time from randomization to death or disease progression. The treatment effect was evaluated according to the Response Evaluation Criteria in Solid Tumors and Guidelines (RECIST) version 1.1, using computed tomography within two weeks before the treatment, at the beginning of the treatment, once per cycle during the first two cycles, and then once every two cycles.

### Statistical analysis

Data analysis was carried out using SAS 9.4 (SAS Institute Inc.). The OS and PFS were analyzed using the Kaplan–Meier method and compared with the log‐rank test. In the prespecified subgroup analysis stratified by hypertension and ECOG score, the univariable proportional hazards (Cox) model was used to estimate the hazard ratios (HRs) for PFS and OS with 95% confidence intervals (CIs). The chi‐square test or Fisher's exact test was used to investigate the correlations between OS or PFS and the stratified variables. Two‐sided *p*‐values <0.05 were considered statistically significant.

## RESULTS

### Patient characteristics

From March 2015 to August 2016, among the patients of the ALTER0303 trial, 53 and 33 patients with SCC received anlotinib and the placebo, respectively. The clinical characteristics of these patients are listed in Table 1.

**TABLE 1:** Clinical characteristics of the SCC patients enrolled in the study

Characteristic	Anlotinib (*n* = 53)	Placebo (*n* = 33)
Age (years), n (%)		
≤60	21 (39.6%)	17 (51.5%)
>60	32 (60.4%)	16 (48.5%)
Sex, n (%)		
Male	48 (90.6%)	30 (90.9%)
Female	5 (9.4%)	3 (9.1%)
Smoking, current, n (%)	47 (88.7%)	30 (90.9%)
Stage, n (%)		
II^a^	2 (3.8%)	0
IIIB	8 (15.1%)	3 (9.1%)
IV	43 (81.1%)	30 (90.9%)
Number of metastases, n (%)		
≤3	38 (71.7%)	22 (66.7%)
>3	15 (28.3%)	11 (33.3%)
ECOG, n (%)		
0	8 (15.1%)	5 (15.2%)
1	44 (83.0%)	28 (84.8%)
2	1 (1.9%)	0
Previous chemotherapy, n (%)		
Pemetrexed	11 (20.8%)	4 (12.1%)
Docetaxel	36 (67.9%)	25 (75.8%)
Paclitaxel	24 (45.3%)	15 (45.5%)
Vinorelbine	13 (24.5%)	6 (18.2%)
Gemcitabine	42 (79.2%)	27 (81.8%)

Abbreviations: ECOG: Eastern Cooperative Oncology Group; SCC: squamous cell carcinoma.

a Postoperative recurrences.

### Stratified analysis according to the occurrence of hypertension

**FIGURE 1:**
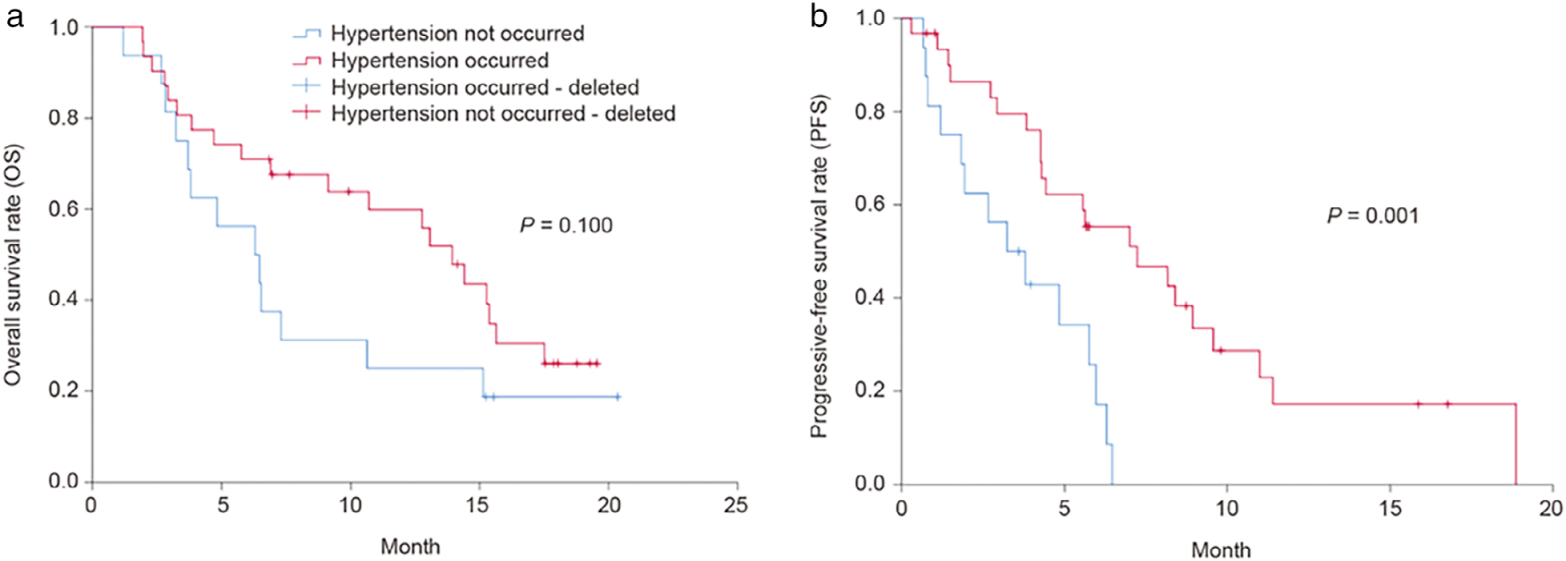
Stratified analysis according to the occurrence of hypertension in patients with squamous cell carcinoma

The 53 SCC patients who received anlotinib were divided into two groups according to the occurrence of hypertension (occurred, *n* = 35; did not occur, *n* = 18). The median OS in the patients was numerically longer if hypertension occurred (13.9 months, 95% CI, 11.5–16.4) compared with those in which it did not occur (6.3 months, 95% CI, 3.1–9.5), but the difference was not significant (*p* = 0.100, HR (95% CI): 0.6 (0.3–1.2)) (Figure 1(a),(b)). The median PFS in the patients who developed hypertension was longer than in those who did not (7.2 (95% CI: 3.5–11.0) vs. 3.2 (95% CI: 1.2–5.3) months, *p* = 0.001; HR (95% CI), 0.4 (0.2–0.8)) (Table 2).

**TABLE 2:** Cox proportional hazards regression analysis for OS and PFS stratified by hypertension

	Hypertension	Median value			Risk		
		Estimated	Standard error	95% CI	HR	95% CI	*p*‐value
OS	Not occurred	6.30	1.63	3.10 to 9.50			
	Occurred	13.93	1.26	11.46 to 16.41	0.56	0.26 to 1.22	0.100
	Total	10.70	3.46	3.91 to 17.49			
PFS	Not occurred	3.23	1.06	1.16 to 5.31			
	Occurred	7.23	1.90	3.51 to 10.96	0.36	0.16 to 0.84	0.001
	Total	5.63	0.84	3.98 to 7.28			

Abbreviations: ECOG: Eastern Cooperative Oncology Group; OS, overall survival; PFS, progression‐free survival.

### Stratified analysis according to the ECOG score

**FIGURE 2:**
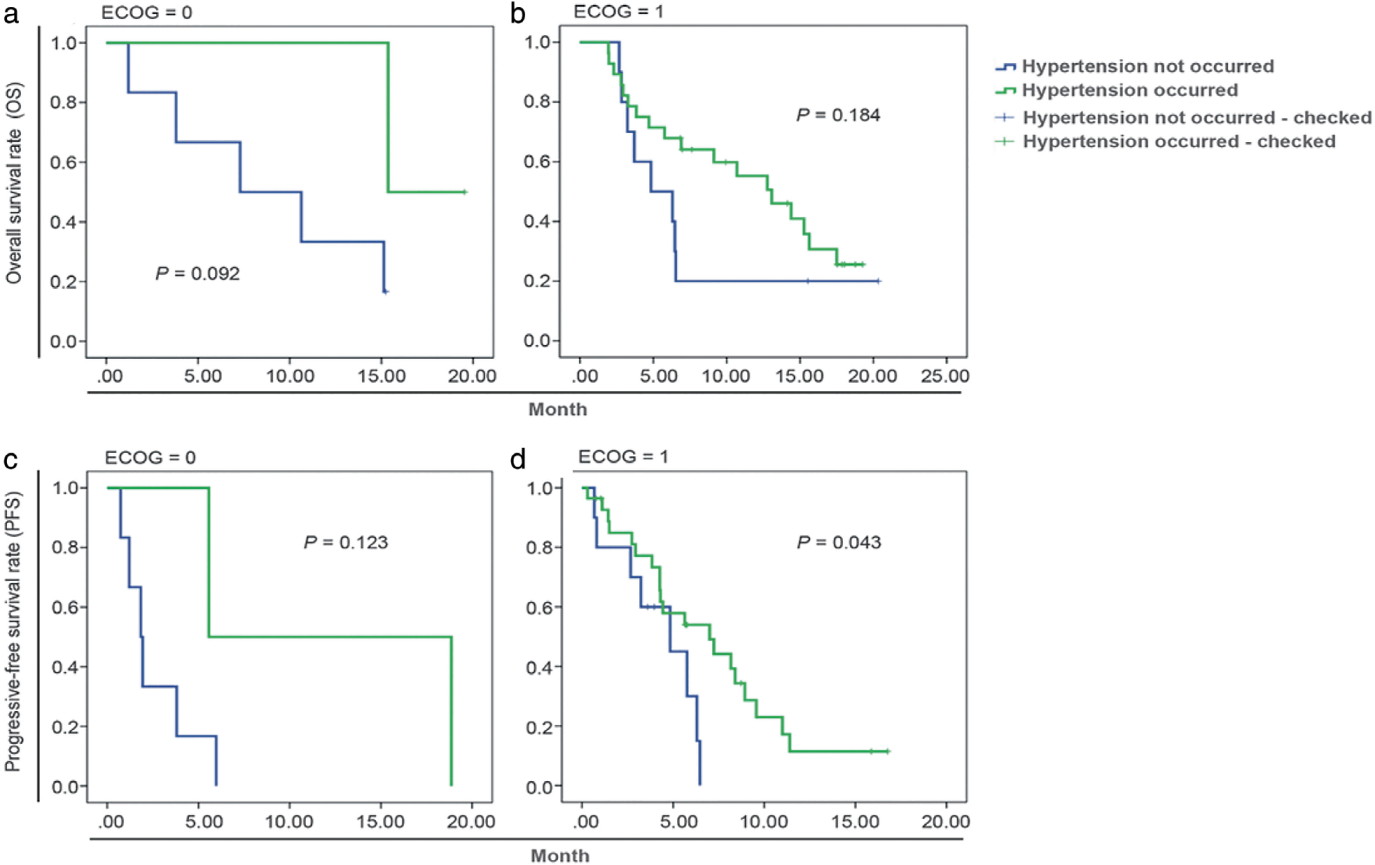
Stratified analysis according to the Eastern Cooperative Oncology Group (ECOG) score in patients with squamous cell carcinoma

Because only one patient with ECOG 2 was enrolled, the SCC patients with ECOG score 0 and 1 were stratified according to the occurrence of hypertension. The median OS of patients who developed hypertension was numerically higher than in those who did not develop hypertension in the ECOG 0 patients (15.4 vs. 7.3 months, *p* = 0.092) and ECOG 1 (13.1 vs. 4.8 months, *p* = 0.184), but the difference was not significantly different (Figure 2(a),(b), Table 3). In the ECOG 0 patients, the median PFS in the patients who developed hypertension versus those who did not was 5.6 vs. 1.8 months, respectively (Figure 2(c)). In the ECOG 1 patients, the median PFS in patients who developed hypertension versus those who did not was 7.0 (95% CI: 3.0–11.0) vs. 4.8 (95% CI, 1.2–8.5) months (*p* = 0.043) (Figure 2(c),(d)).

**TABLE 3:** Cox regression analysis for OS and PFS stratified by ECOG score

		Hypertension	Median value			*p*‐value
			Estimated	Standard error	95% CI	
OS	ECOG = 0	Not occurred	7.30	4.19	0.00 to 15.50	0.092
		Occurred	15.37	–	–	
	ECOG = 1	Not occurred	4.83	2.06	0.81 to 8.86	0.184
		Occurred	13.07	2.61	7.96 to 18.18	
PFS	ECOG = 0	Not occurred	1.83	0.45	—	0.123
		Occurred	5.57	—	—	
	ECOG = 1	Not occurred	4.83	1.86	1.19 to 8.48	0.043
		Occurred	7.00	2.06	2.97 to 11.03	

Abbreviations: ECOG: Eastern Cooperative Oncology Group; OS, overall survival; PFS, progression‐free survival.

## DISCUSSION

There is a lack of targeted therapeutic options for SCC. Accelerated hypertension is an issue with many targeted therapies for lung cancer. This post hoc analysis of the ALTER0303 trial aimed to analyze the efficacy of anlotinib, based on the PFS and OS in patients with SCC, stratified by hypertension and ECOG score. The results suggest that the occurrence of hypertension might be a clinical indicator predicting the efficacy of third‐line anlotinib treatment in patients with SCC.

Platinum‐based chemotherapy is still the dominant therapeutic strategy in patients with SCC, but the prognosis is poor, with a median OS of about 9–11 months for first‐line treatment.^16^, ^27^ Data presented by our team at the 2018 ASCO Annual Meeting in Chicago showed that the median OS in patients with SCC treated with third‐line anlotinib was about 10.7 months, which was not significantly different from that in the placebo group. Nevertheless, those data are still valuable because the efficacy of anlotinib was confirmed in the third‐line and not only in the first. Moreover, the original ALTER0303 study reported that the median OS is about 9.3 months in patients with NSCLC, including 75% of adenocarcinoma.^14^ Thus, anlotinib is possibly comparable in the treatment of SCC and adenocarcinoma. On the other hand, the data in this phase III trial also showed that the median PFS was 5.6 months in patients with SCC treated with anlotinib, which suggests the efficacy of anlotinib in the treatment of SCC because the PFS has been reported to be only 2.3–2.7 months in second‐line treatment with pemetrexed and docetaxel.^28^ Nevertheless, head‐to‐head comparisons of different regimens are necessary to reach firm conclusions.

Anlotinib is an oral multitarget tyrosine kinase inhibitor. Previous studies have shown that hypertension is one of the most common adverse cardiovascular effects in several solid malignancies during treatment with TKIs.^29^ We, therefore, analyzed the efficacy of anlotinib stratified by hypertension and the ECOG score. The median OS in the patients who developed hypertension could be up to 13.1 months. Interestingly, the median OS of patients with SCC treated with anlotinib is similar to that of some novel immunotherapies such as pembrolizumab (OS, 10.4 months),^30^ nivolumab (OS, 9.0 months),^31^ and atezolizumab (OS, 12.6 months).^32^ Nevertheless, the PFS could be significantly extended up to 7.0 months, while it is only about 5.6 months in platinum‐based chemotherapy as first‐ or second‐line therapy.^8^ Therefore, it is reasonable to believe that the occurrence of hypertension could be used as a specific clinical indicator during anlotinib treatment for predicting prognosis in patients with SCC. In the stratified analysis by ECOG score, the median OS was further improved, and the PFS was significantly extended in the subgroup (ECOG 1) of patients who developed hypertension, which may suggest a better tolerance to anlotinib because of the close relationship between physical activity and hypertension.^33^

It has been confirmed that hypertension is the most common adverse reaction (incidence of about 30%–40%) during treatment with antiangiogenic drugs.^34^ Nevertheless, the pathogenetic mechanisms of adverse cardiovascular effects such as hypertension during anlotinib treatment remain elusive. Since about 60% of the malignant tumors express high levels of VEGF, the inhibition of the VEGF signaling pathway should decrease the proper blood supply to the tumor. It is speculated that anti‐VEGF drugs can reduce the production of nitrous oxide by endothelial cells, leading to vasoconstriction, affecting the secretion of sodium by the kidney, and finally leading to increased blood pressure.^23^ Moreover, many angiogenesis inhibitors, such as sunitinib and sorafenib, have off‐target effects and might be the possible causative factors of VEGFR TKI‐related hypertension.^35^ Notably, a previous study reported that antiangiogenesis‐related adverse events might be used as biomarkers for predicting a favorable drug response.^36^ Anlotinib has been proven to have broad‐spectrum antitumor activity in several kinds of cancer.^37^ This post hoc analysis showed that anlotinib‐associated hypertension might indicate a better outcome of SCC patients when cardiovascular toxicity is manageable and acceptable.^38^

Of course, this study has limitations. It was a post hoc analysis of a phase III randomized controlled trial, limited by the small sample size. Because of the small sample size, the hypertension group could not be stratified according to the different grades of CTCAE‐defined hypertension. However, the patients were stratified according to ECOG, even though the number of patients with ECOG 0 was small, and because ECOG is a confounding factor for the adverse events of antiangiogenesis drugs.^26^, ^36^, ^39^ Additional studies with a larger sample size are needed to address this issue. There was a strong possibility of an immortal time bias in the survival analysis. Finally, data about the use of antihypertensive medication was not documented in the original trial. In addition, central SCCs with cavitary features were excluded in the original trial, and this study does not represent all SCCs. Further large sample size trials are needed to verify our findings.

In conclusion, this study shows that hypertension, as an adverse event to anlotinib, might be a valuable indicator for the prognosis of patients with SCC treated with anlotinib. Nevertheless, the prognostic impact of this anlotinib‐induced hypertension remains to be determined, especially for patients with hypertension and/or other cardiovascular diseases at baseline. It could be of clinical relevance, especially in cases with longer‐term therapy. In a future study, the relationship between hypertension and anlotinib efficacy could be verified in the entire population of patients.

## DISCLOSURE

The authors declare that they have no competing interests.
